# Allogeneic stem cell transplantation for AML patients with RUNX1 mutation in first complete remission: a study on behalf of the acute leukemia working party of the EBMT

**DOI:** 10.1038/s41409-021-01322-w

**Published:** 2021-05-31

**Authors:** Johanna Waidhauser, Myriam Labopin, Jordi Esteve, Nicolaus Kröger, Jan Cornelissen, Tobias Gedde-Dahl, Gwendolyn Van Gorkom, Jürgen Finke, Montserrat Rovira, Nicolaas Schaap, Eefke Petersen, Dietrich Beelen, Donald Bunjes, Bipin Savani, Christoph Schmid, Arnon Nagler, Mohamad Mohty, on behalf of the Acute Leukemia Working Party of EBMT

**Affiliations:** 1 Department of Hematology and Medical Oncology, University Medical Center Augsburg, Augsburg, Germany; 2 EBMT Paris Study Office, Saint Antoine Hospital, Paris, France; 3 INSERM UMR 938, Sorbonne University, Paris, France; 4 Institute of Hematology and Oncology, Hospital Clinic Barcelona, Barcelona, Spain; 5 Bone Marrow Transplantation Centre, University Hospital Eppendorf, Hamburg, Germany; 6 Department of Hematology, University Medical Center Rotterdam, Rotterdam, The Netherlands; 7 Clinic for Cancer Medicine, Oslo University Hospital, Rikshospitalet, Oslo, Norway; 8 Department of Internal Med. Hematology /Oncology, University Hospital Maastricht, Maastricht, The Netherlands; 9 Department of Hematology and Medical Oncology, University of Freiburg, Freiburg, Germany; 10 Department of Hematology, Nijmegen Medical Centre, Nijmegen, The Netherlands; 11 Department of Hematology, University Medical Centre Utrecht, Utrecht, The Netherlands; 12 Department of Bone Marrow Transplantation, University Hospital Essen, Essen, Germany; 13 Klinik fuer Innere Medizin III, University Hospital Ulm, Ulm, Germany; 14 Vanderbilt University Medical Center, Nashville, TN USA; 15 Tel Aviv University, BMT and Cord Blood Bank, Chaim Sheba Medical Center, Tel-Hashomer, Israel; 16 Department of Hematology, Saint Antoine Hospital, Paris, France

**Keywords:** Acute myeloid leukaemia, Stem-cell therapies

## Abstract

Acute myeloid leukemia with runt-related transcription factor 1 gene mutation (*RUNX1*+ AML) is associated with inferior response rates and outcome after conventional chemotherapy. We performed a retrospective, registry-based analysis to elucidate the prognostic value of *RUNX1* mutation after allogeneic stem cell transplantation (alloSCT). All consecutive adults undergoing alloSCT for AML in first complete remission (CR1) between 2013 and 2019 with complete information on conventional cytogenetics and *RUNX1* mutational status were included. Endpoints of interest were cumulative relapse incidence, non-relapse mortality, overall and leukemia-free survival (OS/LFS), and GvHD-free/relapse-free survival. A total of 674 patients (183 *RUNX1*+, 491 *RUNX1*−) were identified, with >85% presenting as *de novo* AML. Median follow-up was 16.4 (*RUNX1*+) and 21.9 (*RUNX1*−) months. Survival rates showed no difference between *RUNX1*+ and *RUNX1*− patients either in univariate or multivariate analysis (2-year OS: 67.7 vs. 66.1%, *p* = 0.7; 2-year LFS: 61.1 vs. 60.8%, *p* = 0.62). Multivariate analysis identified age, donor type and poor cytogenetics as risk factors for inferior outcome. Among patients with *RUNX+* AML, older age, reduced intensity conditioning and minimal residual disease at alloSCT predicted inferior outcome. Our data provide evidence that the negative influence of *RUNX1* mutations in patients with AML can be overcome by transplantation in CR1.

## Introduction

Increasing knowledge about the impact of runt-related transcription factor 1 (*RUNX1*) gene mutation on characteristics and outcome of patients with *de novo* acute myeloid leukemia (AML) has led to its establishment as a provisional entity in the 2016 WHO classification [1]. In contrast to diseases with cytogenetic alterations leading to *RUNX* transcription factor family rearrangement such as t(8;21)(q22;q22), AML with *RUNX1* gene mutations is associated with poorer response to conventional chemotherapy, lower rates of complete remission (CR), relapse free survival (RFS), and overall survival (OS) [2–8]. These findings led to the allocation of *RUNX1* mutated AML to the adverse risk category of the 2017 European Leukemia Net (ELN) risk stratification [9]. However, data on the relevance of this mutation to the indication for and outcome after allogeneic stem cell transplantation (alloSCT) are scarce and contradictory and came mainly from subgroup analyses containing low numbers of transplant recipients. Schnittger et al. did not observe any survival advantage after alloSCT among 17 out of 97 *RUNX1*+ patients who underwent alloSCT in first CR or as salvage therapy [4]. In contrast Gaidzik et al. found a better RFS after transplantation in subgroup analyses of two different studies on *RUNX1*+ patients, including 14/53 and 36/245 patients undergoing alloSCT, but without an impact of the mutation on survival among allografted patients [5, 7].

Here, we present the results of a large retrospective study on 674 patients, performed by the Acute Leukemia Working Party (ALWP) of the European Society for Blood and Marrow Transplantation (EBMT) and designed specifically to analyze the prognostic value of *RUNX1* mutation after allo SCT for AML.

## Methods

### Patients

Data were extracted from the EBMT registry. The EBMT is a non-profit, scientific society representing more than 600 transplant centers, that are required to report all consecutive stem cell transplantations including annual follow-up. Data are managed in a central database with internet access, annual audits are performed to verify data accuracy. Patients provide informed consent authorizing the use of their personal information for research purposes before transplantation.

This study was approved by the by the general assembly of the ALWP of the EBMT. We screened the EBMT database for adult AML patients with complete information on cytogenetics and *RUNX1* mutational status, excluding patients with translocation t(8;21)/*RUNX1*−*RUNX1T1*. Further inclusion criteria were an alloSCT in CR1 between January 2013 and June 2019 from either a matched sibling donor (MSD), matched unrelated donor (MUD, with a minimum of 9/10 HLA match) or haploidentical donor (T-cell replete haplo-SCT only). Intensity of conditioning therapy (myeloablative conditioning; MAC vs. reduced-intensity conditioning; RIC), cytomegalovirus (CMV) status, T-cell depletion of transplant, and Karnofsky Perfomance Score (KPS), data on co-mutations such as nucleophosmin 1 (*NPM1*), FMS-like tyrosine kinase 3 internal tandem duplication (*FLT3*-ITD) and additional sex combs-like 1 (*ASXL1*) were retrieved from the registry when available, as was minimal residual disease (MRD) status at time of alloSCT (evaluated according to local standards). Outcome variables were OS and leukemia-free survival (LFS), graft-versus-host disease GvHD-free/relapse-free survival (GRFS), cumulative relapse incidence (RI), non-relapse mortality (NRM) and GvHD.

### Definitions and statistical analysis

OS was defined as the time interval between dates of transplantation and death from any cause, LFS as the interval between dates of transplantation and relapse, death in remission or last follow up. Cytogenetic subgroups, acute GvHD (aGvHD) and chronic GvHD (cGvHD) were classified as previously defined [10–12], the composite endpoint GRFS was defined as survival after transplantation without aGvHD grade III–IV, or cGvHD with indication for treatment, or relapse [13]. RI was defined as the period between transplantation and disease progression, NRM as death from any cause without relapse or progression of the initially diagnosed leukemia.

Patient, disease, and transplant-related characteristics of the two cohorts (*RUNX1+* and *RUNX1*−) were compared by using the χ^2^ or Fisher’s exact test for categorical variables, and the Mann-Whitney test for continuous variables. For univariate analysis, continuous variables were categorized, and the median value used as a cut-point; the log-rank test and Gray’s test were used for comparison of outcome between groups. The date of transplantation was the starting point for time-to-event analyses. Survivors were censored at last contact. Cumulative incidence was used to estimate the endpoints of NRM, RI, aGvHD and cGVHD, accommodating for competing risks. A Cox proportional-hazards model was used for multivariate regression; all factors shown to be significant at the *p* < 0.05 in the univariate analysis, or those known from the literature to possibly influence outcome were included. A random effect (frailty effect) was introduced into Cox models to consider the heterogeneity in the effect of a characteristic or a treatment across centers. Multivariate results are expressed as a hazard ratio (HR) with a 95% confidence interval (CI). All tests were two sided. The type-1 error rate was fixed at 0.05 for determination of factors associated with time-to-event outcomes. All analyses were performed using SPSS 24.0 (SPSS Inc, Chicago, IL, USA)) and R version 3.4.0 (R Core Team. R: a language for statistical computing. 2014. R Foundation for Statistical Computing, Vienna, Austria).

## Results

### Patient and disease characteristics

A total of 674 patients (183 (27%) with and 491 (73%) without *RUNX1* mutation) were included. The median age was 57 (range 18–77) years, 302 (45%) patients were female. AML was classified as *de novo* disease in 88 and 83% of *RUNX1*− and *RUNX1*+ patients, respectively. Most patient, disease, and transplant characteristics were equally distributed between the two cohorts, including MRD status before SCT (Table 1). However, as expected [3, 6, 14, 15], an imbalance in the frequency of *NPM1* and *ASXL1* mutations was observed. Thus, *NPM1* mutation was detected in 26% of *RUNX1*− patients but in only 5% of *RUNX1*+ patients (*p* < 0.0001). In contrast, an *ASLX1* mutation was more frequent in *RUNX1*+ patients than in those lacking this mutation (55 vs. 16%, *p* < 0.0001). *FLT3*-ITD was observed at a similar frequency (26 vs. 30%) in both groups.

**Table 1:** Demographic and disease characteristics of total population and *RUNX1*+ and *RUNX1*− subgroups.

Variables	Total	RUNX1−	RUNX1+	*p* value
**Age**; *median (range)*	57.4 (18.2–77.4)	56.8 (18.3–77.4)	58.7 (18.2–75)	0.067
**Sex**				0.16
Male; *n (%)*	372 (55.2)	279 (56.8)	93 (50.8)	
Female; *n (%)*	302 (44.8)	212 (43.2)	90 (49.2)	
**deNovo AML**; *n (%)*	584 (86.6)	432 (88)	152 (83.1)	0.095
**Cytogenetics**				0.62
Intermediate; *n (%)*	510 (75.7)	374 (76.2)	136 (74.3)	
Poor; *n (%)*	164 (24.3)	117 (23.8)	47 (25.7)	
**Donor type**				0.18
MSD; *n (%)*	169 (25.1)	121 (24.6)	48 (26.2)	
MUD; *n (%)*	451 (66.9)	336 (68.4)	115 (62.8)	
Haplo; *n (%)*	54 (8.0)	34 (6.9)	20 (10.9)	
**KPS**				0.93
<90; *n (%)*	197 (30.6)	142 (30.5)	55 (30.9)	
>90; *n (%)*	446 (69.4)	323 (69.5)	123 (69.1)	
**CMV recipient serostatus**				0.57
Positive; *n (%)*	424 (63.3)	305 (62.6)	119 (65)	
Negative; *n (%)*	246 (36.7)	182 (37.4)	64 (35)	
**T-cell depletion**				0.09
Yes; *n (%)*	407 (60.8)	297 (61)	110 (60.4)	
No; *n (%)*	262 (39.2)	190 (39)	72 (39.6)	
**Intensity of conditioning**				0.099
RIC; *n (%)*	359 (53.9)	252 (52)	107 (59.1)	
MAC; *n (%)*	307 (46.1)	233 (48)	74 (40.9)	
**NPM1**				**<0.0001**
Positive; *n (%)*	126 (22.9)	121 (26.4)	5 (5.4)	
Negative; *n (%)*	425 (77.1)	338 (73.6)	87 (94.6)	
Missing; *n*	123	32	91	
**ASXL1**				**<0.0001**
Positive; *n (%)*	67 (26)	30 (15.7)	37 (55.2)	
Negative; *n (%)*	191 (74)	161 (84.3)	30 (44.8)	
Missing; *n*	416	300	116	
**FLT3-ITD**				0.43
*Positive; n (%)*	149 (26.6)	119 (25.9%)	30 (29.7%)	
*Negative; n (%)*	412 (73.4)	341 (74.1%)	71 (70.3%)	
*Missing; n*	113	32	91	
**MRD at HSCT**				0.13
Positive; *n (%)*	123 (40.6)	90 (43.5)	33 (34.4)	
Negative; *n (%)*	180 (59.4)	117 (56.5)	63 (65.6)	
Missing; *n*	371	284	87	
**Median follow up***(months)*	19.6	21.9	16.4	**0.004**

*MSD* matched sibling donor, *MUD* matched unrelated donor, *KPS* Karnofsky performance score, *CMV* cytomegalovirus, *RIC* reduced intensity conditioning, *MAC* myeloablative conditioning. Statistically significant p-values are in bold.

### Overall outcome and univariate analysis

Median follow up from SCT was 19.6% (21.9 months among RUNX-, 16.4 months among RUNX+ patients, *p* = 0.004). Within the entire cohort, cumulative RI and NRM were 23.8% (95% CI: 20.2–27.6) and 15.2% (95% CI: 12.4–18.4), OS, LFS, and GRFS, at 2 years from transplantation were 66.6% (95% CI: 62.4–70.8), 61% (95% CI: 56.7–65.3), and 45.5% (95% CI: 41.2–49.8), respectively (Table 2). Cumulative incidence of acute GvHD (aGvHD) by day 180 was 28% (95% CI: 24.6–31.6) for grades II–IV, and 9.1% (95% CI: 7.1–11.5) for grades III–IV. At 2 years, cumulative incidence of chronic GvHD (cGvHD) was 37.8% (95% CI: 33.5–42.1) overall and 17.4% (95% CI: 14.1–21) for extensive cGvHD. At 2 years, 189 patients had died. AML was the most frequent cause of death, accounting for 35% of all fatalities, followed by infections (24%) and GvHD (15%).

**Table 2:** Overall outcome and multivariate analysis of relevant factors for outcome among 674 patients undergoing alloSCT in CR1 for AML with (*n* = 183) or without (*n* = 491) *RUNX1* mutation.

Entire cohort (*n* = 674)										
**Overall outcome**	**RELAPSE**		**NRM**		**LFS**		**OS**		**GRFS**	
	23.8% (95% CI: 20.2–27.6)		15.2% (95% CI: 12.4–18.4)		61% (95% CI: 56.7–65.3)		66.6% (95% CI: 62.4–70.8)		45.5% (95% CI: 41.2–49.8)	
**Multivariate analysis**	**HR (95% CI)**	***p*value**	**HR (95% CI)**	***p*value**	**HR (95% CI)**	***p*value**	**HR (95% CI)**	***p*value**	**HR (95% CI)**	***p*value**
*RUNX1* mutation (present vs. absent)	1.05 (0.66–1.69)	0.83	0.65 (0.32–1.32)	0.23	0.9 (0.61–1.33)	0.6	0.91 (0.59–1.39)	0.65	1.15 (0.84–1.57)	0.38
Donor type (UD/Haplo vs. MSD)	1.09 (0.71–1.68)	0.69	2.17 (1.08–4.39)	**0.03**	1.37 (0.95–1.97)	0.092	1.56 (1.04–2.35)	**0.031**	1.13 (0.84–1.52)	0.42
Age (per 10y)	0.92 (0.79–1.06)	0.26	2.07 (1.54-2.78)	**<0.0001**	1.14 (1–1.3)	0.054	1.27 (1.09-1.48)	**0.002**	1.05 (0.94–1.17)	0.37
AML presentation (secondary vs. de novo)	1.89 (1.18-3.01)	**0.008**	0.51 (0.21–1.22)	0.13	1.29 (0.86–1.94)	0.22	1.05 (0.66-1.66)	0.85	1.08 (0.75–1.55)	0.68
Cytogenetic risk (poor vs. intermediate)	2 (1.34-2.99)	**0.0007**	1.49 (0.86–2.6)	0.16	1.82 (1.32–2.52)	**0.0003**	1.68 (1.18–2.39)	**0.004**	1.63 (1.24–2.15)	**0.0005**
*FLT3* ITD(present vs. absent)	1.64 (1.1–2.46)	**0.016**	0.97 (0.54–1.77)	0.93	1.34 (0.96-1.87)	0.085	1.24 (0.86–1.78)	0.26	1.44 (1.1–1.89)	**0.008**
Donor-recipient gender (female to male vs. other)	0.95 (0.54–1.69)	0.87	2.02 (1.15–3.56)	**0.015**	1.31 (0.88–1.95)	0.18	1.37 (0.9–2.08)	0.14	1.08 (0.76–1.53)	0.66
Conditioning (RIC vs. MAC)	1.42 (0.95–2.13)	0.091	0.94 (0.54–1.65)	0.84	1.27 (0.91–1.76)	0.16	1.26 (0.88–1.81)	0.21	1.1 (0.82–1.47)	0.51
In vivo TCD	1.18 (0.8–1.73)	0.4	0.63 (0.38–1.04)	0.072	0.92 (0.68–1.25)	0.61	0.8 (0.58–1.12)	0.19	0.74 (0.56–0.98)	**0.036**

*NRM* non-relapse mortality, *LFS* leukemia-free survival, *OS* overall survival, *GRFS* GvHD-free/relapse-free survival, *MUD* matched unrelated donor, *MSD* matched sibling donor, *MAC* myeloablative conditioning, *RIC* reduced intensity conditioning, *TCD* T-cell depletion. Statistically significant p-values are in bold.

**Fig. 1:**
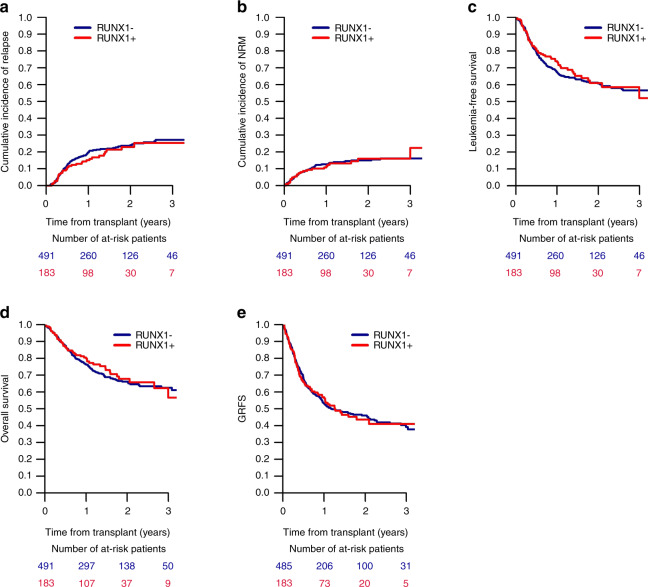
Outcome of 674 patients with AML with or without RUNX1 gene mutation transplanted in first CR. **a** cumulative relapse incidence (RI), **b** non-relapse mortality (NRM), **c** leukemia-free survival (LFS), **d** overall survival (OS), **e** GvHD-free/relapse-free survival (GRFS).

In univariate analysis, risk factors for inferior OS and LFS were poor cytogenetics, older age and RIC. Cytogenetics and secondary AML (sAML) were risk factors for RI, NRM was influenced by cytogenetics, donor type, age and conditioning, and GRFS by cytogenetics and *FLT3*-ITD. In contrast, presence of the *RUNX1* mutation did not influence any of the investigated outcomes (RI, NRM, LFS, OS, and GRFS; Supplementary Table 1 and Fig. 1). Similarly, no influence of the *RUNX1* mutation on any outcome parameter could be demonstrated, when the analysis was limited to 584 patients (87%) with *de novo* AML or to 510 patients (76%) with intermediate cytogenetics (Supplementary Table 2, Supplementary Figs. 1 and 2).

An exploratory analysis was performed within the cohorts of patients with available data regarding *ASXL1* co-mutation, since a significant interaction between these two mutations, leading to a particularly poor prognosis, had been suggested [14]. However, among 258 patients with available data on both markers (38% of the entire cohort), the existence of both mutations was not a prognostic indicator of any outcome after allo-SCT (Supplementary Table 3).

### Multivariate analysis

In a multivariate analysis of the entire cohort, sAML, poor cytogenetics, and *FLT3*-ITD negatively affected RI. NRM was associated with donor type other than HLA identical family donor, female donor to male patient, and older age. Donor type, older age and poor risk cytogenetics showed a negative impact on OS, poor risk cytogenetics negatively affected LFS. Factors affecting GRFS were cytogenetic category, *FLT3*-ITD and in vivo T-cell depletion. In contrast, *RUNX1* mutational status did not show a significant influence on any outcome (Table 2). Again, similar results were obtained when the analysis was limited to patients with *de novo* AML. A third multivariate model was fitted for the 510 patients with intermediate cytogenetics (*RUNX1*−, *n* = 374, and *RUNX1+*, *n* = 136). Even in this subgroup, *RUNX1* mutation was not associated with any outcome, whereas older age was the main factor associated with lower OS. RI was influenced by *FLT3*-ITD and RIC, NRM was influenced by older age, donor type and a female donor for male recipient (Supplementary Table 4).

### Prognostic factors among patients bearing a *RUNX1* mutation

To further characterize this cohort, a risk factor analysis was performed among patients with AML and *RUNX1* mutation. In univariate analysis, OS and LFS were influenced by cytogenetics, age (borderline significance for LFS), conditioning, MRD at time of transplantation and AML type (*de novo* versus secondary). NRM was influenced by age and MRD status, whereas conditioning and AML subtype influenced RI. In the multivariate model, both inferior OS and LFS were significantly associated with older age, RIC, and MRD positivity. Unfortunately, the number of events was too low for a multivariate analysis of RI and NRM (Table 3).

**Table 3:** Analysis of risk factors for outcome after allogeneic SCT among patients with AML bearing a *RUNX1* mutation in first complete remission. (A) Univariate analysis, (B) Multivariate analysis (*n* = 183).

(A) Univariate analysis							
			2 years				
		*N* (%)	RI	NRM	LFS	OS	GRFS
**Cytogenetic risk**	Intermediate	136 (74.3%)	21.5% [13.2–31.1]	13.6% [7.5–21.5]	64.9% [53.4–74.3]	73.9% [63.1–82]	46.6% [35.8–56.7]
	Poor	47 (25.7%)	26.5% [13.8–40.9]	23.1% [10.4–38.8]	50.4% [32.8–65.7]	51.4% [32.7–67.3]	35% [19.7–50.8]
	*P* value		0.08	0.06	**0.003**	**0.006**	**0.024**
**Donor**	MSD	48 (26.2%)	24.6% [10.9–41.2]	13.2% [3.2–30.5]	62.2% [40.7–77.8]	56% [33–73.9]	40.6% [24.7–55.9]
	MUD	115 (62.8%)	22.4% [13.7–32.4]	16.7% [9.8–25.2]	61% [49.1–70.8]	71.8% [60.8–80.2]	43.9% [32.6–54.6]
	Other relative	20 (10.9%)	24% [5.2–50.2]	19.4% [3.7–44.4]	56.6% [26.2–78.5]	63.6% [31.7–83.7]	53.4% [24.8–75.4]
	*P* value		0.95	0.67	0.87	0.82	0.62
**In vivo T-cell depletion**	No TCD	72 (39.6%)	21.4% [11.6–33.2]	17.5% [9–28.3]	61.1% [46.8–72.6]	64.7% [49.9–76.1]	37.2% [24.8–49.7]
	TCD	110 (60.4%)	23.9% [13.6–35.7]	15.4% [7.7–25.5]	60.7% [46.5–72.2]	71.5% [58.7–81]	48.2% [35.2–60]
	*P* value		0.65	0.39	0.76	0.34	0.12
**Patient age**	Age<median	91 (50%)	22.9% [13.4–34.1]	9.8% [4–18.7]	67.2% [53.9–77.5]	73.5% [60.3–82.9]	40% [27.8–51.9]
	Age>median	92 (50%)	23.1% [12.8–35.2]	22.9% [13.1–34.3]	54% [39.6–66.4]	61% [46.3–72.9]	46.8% [33.6–58.9]
	*P* value		0.88	**0.015**	0.09	**0.034**	0.44
**Conditioning**	MAC	74 (40.9%)	14.9% [6.5–26.4]	10.8% [4.6–20]	74.3% [60.1–84.1]	78% [64.4–86.9]	52.4% [38.6–64.5]
	RIC	107 (59.1%)	29.4% [18.9–40.7]	20.6% [11.6–31.4]	50% [36.8–61.8]	59.1% [45.6–70.4]	36.4% [24.6–48.3]
	*P* value		**0.048**	0.15	**0.007**	**0.016**	0.19
**MRD**	MRD neg	63 (65.6%)	16.5% [6.7–30.2]	10% [2.4–24]	73.5% [54.8–85.4]	77.6% [57.5–89]	53.6% [36.5–68]
	MRD pos	33 (34.4%)	30.1% [12.9–49.3]	23.2% [9.8–39.9]	46.8% [26.3–64.9]	60.3% [38.8–76.3]	37.8% [19.2–56.3]
	*P* value		0.2	**0.043**	**0.008**	**0.046**	0.11
**AML type**	De novo	152 (83.1%)	18% [11.4–25.9]	15.5% [9.1–23.4]	66.5% [56.2–74.9]	71.2% [60.6–79.5]	48.6% [38.9–57.7]
	SecAML	31 (16.9%)	42.3% [20.6–62.6]	19.7% [6.8–37.3]	38% [18.1–57.8]	51.3% [29.8–69.3]	21.8% [6.8–42.2]
	*P* value		**0.023**	0.7	**0.021**	**0.026**	0.051

(B) Multivariate analysis				
	LFS		OS	
	HR (95% CI)	*p* value	HR (95% CI)	*p* value
Age (per 10 years)	**0.7 (0.51**–**0.94)**	**0.02**	**0.65 (0.47**–**0.9)**	**0.008**
Poor vs. intermediate-risk cytogenetics	1.9 (0.77–4.72)	0.17	1.63 (0.61–4.38)	0.33
RIC vs. MAC	**3.99 (1.54**–**10.32)**	**0.004**	**4.56 (1.5**–**13.86)**	**0.007**
Secondary vs. de novo AML	1.38 (0.53–3.6)	0.51	1.53 (0.55–4.29)	0.42
MRD (pre transplant) positivity	**4.26 (1.75**–**10.33)**	**0.001**	**3.63 (1.37**–**9.57)**	**0.009**
Frailty (ID)		0.95		0.96

*LFS* leukemia-free survival, *OS* overall survival, *MAC* myeloablative chemotherapy, *RIC* reduced intensity chemotherapy, *MRD* minimal residual disease. Statistically significant p-values are in bold.

## Discussion

In the last WHO classification of myeloid malignancies, a provisional entity of AML with mutated *RUNX1* was added for patients diagnosed with *de novo* AML containing this mutation and not harboring defining diagnostic criteria for other AML subtypes. This new provisional disease category was considered as a biologically distinct group with inferior prognosis. Moreover, most studies which have analyzed the prognostic value of *RUNX1* mutation on prognosis have observed a negative impact [3–7]. Accordingly, the ELN classification has included *RUNX1* mutation within the adverse risk category. However, in contrast to other genetic aberrations [16, 17], information on the role of *RUNX1* mutation on outcome after alloSCT is scarce. Therefore, the EBMT transplant registry was used for a large-scale retrospective analysis of patients with AML and known mutational status of the *RUNX1* gene, who underwent alloSCT in CR1. Within this population, we did not observe a negative impact of *RUNX1* mutation on any outcome parameter, although follow up was shorter among RUNX+ patients. This was true within the entire cohort, and also in focused analysis among patients with *de novo* AML (>85% of the cohort), and among patients with intermediate-risk cytogenetics. The role of classical risk factors on outcome was not modified by the presence of a *RUNX1* mutation. Within the cohort of *RUNX1*+ patients, age, intensity of conditioning and MRD status before transplantation were associated with outcome after alloSCT in CR1. Hence, optimal disease control and use of myeloablative conditioning might be ways to improve outcome in this particular AML subtype.

Unlike other studies on the role of *RUNX1* mutation on the outcome after alloSCT, this analysis has mainly included patients with *de novo* AML, which all had been transplanted in CR1. In contrast to our results, an association of the *RUNX1* mutation with inferior outcome after allogeneic transplantation was found among patients transplanted for MDS or sAML [18]. This difference might be explained by the inclusion of both diseases, and of patients transplanted in different disease status (CR, active disease) in the latter study. However, compared to de novo disease, *RUNX1* mutations might also have different clinical consequences in MDS and sAML [19, 20], as these diseases comprise higher rates of additional mutations such as *ASXL1*, *TET2*, and *EZH2* [21], leading to the classification of sAML as a separate biological entity by several researchers [22]. This was supported recently by the finding that sAML is an independent risk factor for lower OS and LFS after alloSCT, as compared to *de novo* AML [23].

MRD status has been proven to be a strong predictor for outcome both after conventional induction therapy [24] and allogeneic transplantation [25, 26], regardless of MRD methodology and threshold [27]. Although MRD measurement techniques were heterogenous and followed local standards, this was also observed in our study, where MRD positivity at time of alloSCT was associated with inferior LFS, OS and GRFS among patients with RUNX1 mutation (Table 3). Unfortunately, missing data on MRD status in a relevant number of patients prevented the inclusion of MRD status in the multivariate model of the entire study population. However, since there was no association between MRD and *RUNX1* mutational status (Table 1), it is at least unlikely that differences in MRD status at time of alloSCT have introduced a bias into the comparison between *RUNX1+* and *RUNX1*− cohorts.

Regarding co-mutations of *RUNX1, FLT3-*ITD was observed with similar frequency among *RUNX1*+ and *RUNX1*− patients, which is consistent with prior findings [4]. Whereas the previously described negative prognostic influence of *FLT3*-ITD [28] was reflected in our study by a higher RI and shorter GRFS, there was no mutual interaction of *FLT3*-ITD and *RUNX1* mutation with respect to outcome. As expected, the presence of *ASXL1* mutation was more frequent in *RUNX1*+ than *RUNX1*− patients [14]. The coexistence of *RUNX1* and *ASXL1* mutations has been described as a strong adverse prognostic factor in two series of AML patients [14, 29]. However, in our study, *ASXL1* as single factor did not have any influence on outcome in univariate analysis, nor did the different combinations of *RUNX1* and *ASXL1* mutational status (cf. Supplementary Table 3). This finding is in line with an extensive analysis by Schnittger et al., who could not detect a functional interaction between the two mutations [4]. Hence, our data might indicate that allogeneic transplantation could also have a positive effect on the otherwise negative prognosis in *RUNX1+/ASXL1+* patients. However, since *ASXL1* mutational status could not be included into the multivariate model, this observation must be interpreted with caution and warrants confirmation in a larger cohort.

In summary, this study is the largest analysis focusing specifically on the relevance of *RUNX1* mutational status on the outcome of allogeneic SCT performed in CR1. Since *RUNX1* mutations were not associated with inferior outcome, the data might suggest that the negative influence of *RUNX1* mutations in patients with AML can be overcome by early transplantation. This may be especially true for *de novo* disease, representing >85% of our cohort. Extensive molecular profiling is warranted to further investigate the interaction between different mutations and their influence on outcome after alloSCT.

## Supplementary information

Supplement

## Data Availability

Source data are stored in the ALWP/EBMT database and can be shared upon personal request.
