# Supplementary material for: Allogeneic stem cell transplantation for AML patients with RUNX1 mutation in first complete remission: a study on behalf of the acute leukemia working party of the EBMT
Source: Bone Marrow Transplant. 2021 May 31;56(10):2445–53. doi: 10.1038/s41409-021-01322-w (PMC8486660; doi:10.1038/s41409-021-01322-w)
Supplement: Supplementary file 1 — Supplement [file 41409_2021_1322_MOESM1_ESM.pdf]

Supplementary figure 1 **Outcome of 584 patients with *de novo* AML with or without *RUNX1* gene mutation transplanted in first CR**
a) cumulative relapse incidence (RI), **b)** non-relapse mortality (NRM), **c)** leukemia-free survival (LFS) **d)** overall survival (OS), **e)** GvHD-free/relapse-free survival (GRFS)

Supplementary figure 2 **Outcome of 510 patients with AML and intermediate risk cytogenetics with or without *RUNX1* gene mutation transplanted in first CR**
a) cumulative relapse incidence (RI), **b)** non-relapse mortality (NRM), **c)** leukemia-free survival (LFS) **d)** overall survival (OS), **e)** GvHD-free/relapse-free survival (GRFS)

Supplementary table 1 **Univariate analysis of relevant factors for outcome (entire cohort, n=674);** NRM: non-relapse mortality, RI: relapse incidence, LFS: leukemia-free survival, OS: overall survival, GRFS: GvHD-free/relapse-free survival, MUD: matched unrelated donor, MSD: matched sibling donor, MAC: myeloablative chemotherapy, RIC: reduced intensity chemotherapy, MRD: minimal residual disease, TCD: T cell depletion.

Supplementary table 2 **Outcome among patients with *de novo* AML (n=584) and patients with intermediate-risk cytogenetics (n= 510) as of *RUNX1* mutational status;** RI: relapse incidence, NRM: non-relapse mortality LFS: leukemia-free survival, OS: overall survival, GRFS: GvHD-free/relapse-free survival.

Supplementary table 3 **Exploratory analysis of the prognostic impact of *ASXL1* mutation** (patients with available information); RI: relapse incidence, NRM: non-relapse mortality, LFS: leukemia-free survival, OS: overall survival, GRFS: GvHD-free/relapse-free survival, MRD: minimal residual disease.

Supplementary table 4 **Multivariate analysis among patients with *de novo* AML (n=585) and patients with intermediate-risk cytogenetics (n=510)** RI: relapse incidence, NRM: non-relapse mortality LFS: leukemia-free survival, OS: overall survival, GRFS: GvHD-free/relapse-free survival.

S1a

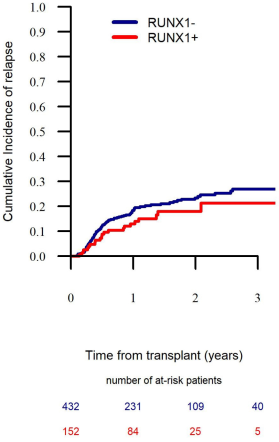

S1b

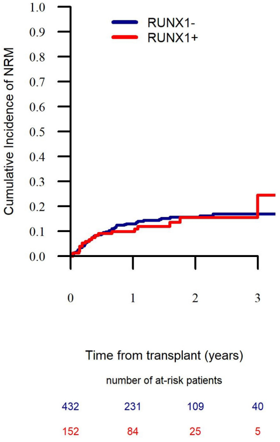

S1c

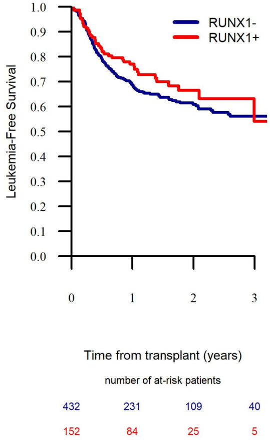

S1d

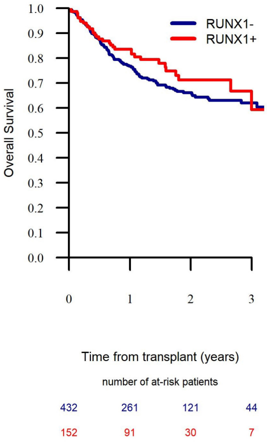

S1e

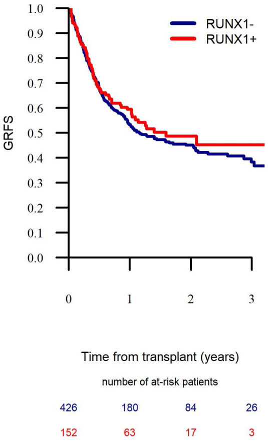

S2a

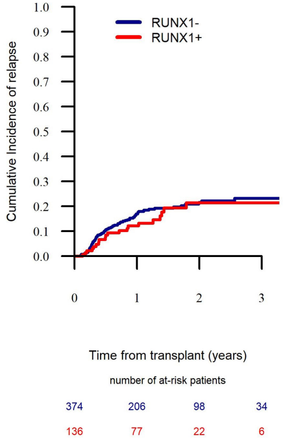

S2b

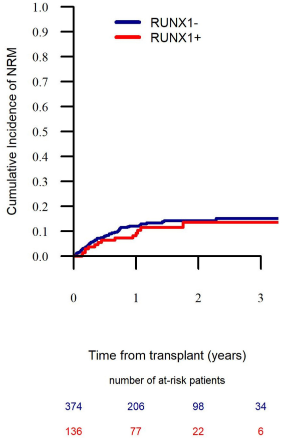

S2c

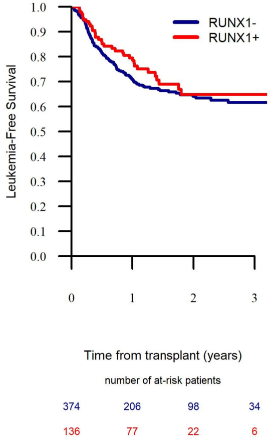

S2d

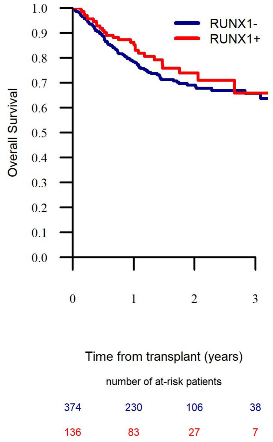

S2e

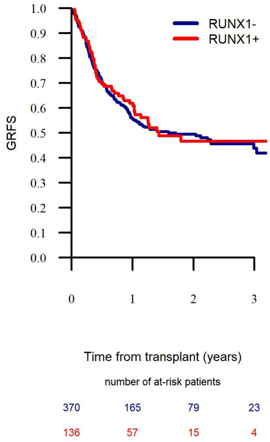

Supplementary Table 1 **Univariate analysis of relevant factors for outcome (entire cohort, n=674)**

**Univariate analysis**						
		**2 years**				
		**RI**	**NRM**	**LFS**	**OS**	**GRFS**
**RUNX1**	Absent	24.2%[20-28.5]	15%[11.8-18.7]	60.8%[55.7-65.5]	66.1%[61-70.7]	46%[41.1-50.9]
	Present	22.9%[15.8-30.9]	16%[10.2-23]	61.1%[51.5-69.3]	67.7%[58.3-75.5]	43.6%[34.6-52.2]
	P value	0.53	0.91	0.62	0.7	1
**Cytogenetics**	intermediate	21.4%[17.4-25.7]	14.1%[10.9-17.6]	64.6%[59.5-69.2]	70.3%[65.4-74.8]	48.9%[43.9-53.8]
	poor	31.2%[23.5-39.1]	18.9%[12.7-25.9]	49.9%[41-58.2]	55.3%[46-63.7]	35%[26.7-43.4]
	P value	**0.001**	**0.08**	**0.001**	**0.001**	**0.001**
**Donor**	MSD	22.6%[15.7-30.3]	9%[4.8-14.8]	68.4%[59.4-75.9]	71.1%[61.5-78.7]	48.6%[39.6-56.9]
	MUD	24.7%[20.3-29.3]	16.7%[13.1-20.7]	58.6%[53.3-63.6]	64.4%[59-69.2]	43.7%[38.5-48.8]
	TR Haplo	19.5%[7.3-35.9]	23.3%[11.6-37.3]	57.3%[38.4-72.3]	70.7%[53.5-82.6]	53.8%[37.5-67.6]
	P value	0.72	**0.036**	0.13	0.13	0.51
**In vivo T-cell depletion**	no TCD	20.9%[15.6-26.8]	18.4%[13.4-23.9]	60.7%[53.5-67.2]	65%[57.8-71.3]	39.2%[32.5-45.8]
	TCD	25.5%[20.7-30.5]	13.4%[10-17.3]	61.2%[55.4-66.4]	67.9%[62.2-72.9]	49.4%[43.7-54.9]
	P value	0.14	0.11	0.95	0.39	**0.01**
**De novo vs. secondary AML**	De novo AML	22.1%[18.4-26.2]	15.5%[12.4-18.9]	62.4%[57.6-66.7]	67.4%[62.7-71.7]	45.9%[41.2-50.5]
	Sec. AML	34.1%[23.3-45.1]	13.9%[7.3-22.6]	52%[39.9-62.8]	61.3%[48.9-71.5]	42.9%[31.4-53.8]
	P value	0.037	0.64	0.14	0.29	0.64
**Patient age**	Age < median	27.4%[22-33.1]	6.6%[4.2-9.9]	65.9%[59.7-71.4]	72.8%[66.6-78]	47.1%[40.8-53.1]
	Age > median	20.1%[15.5-25.2]	23.9%[18.9-29.1]	56%[49.7-61.9]	60.2%[53.8-66.1]	43.9%[37.9-49.8]
	P value	0.08	**0.001**	**0.005**	**0.001**	0.16
**Conditioning**	MAC	23.6%[18.2-29.4]	10.3%[7-14.3]	66.2%[59.6-71.9]	72.7%[66.2-78.1]	48.4%[41.8-54.7]
	RIC	24.2%[19.4-29.2]	19.3%[15-24.1]	56.5%[50.4-62.1]	61.4%[55.2-67]	43.4%[37.6-49.1]
	P value	0.71	**0.006**	**0.019**	**0.001**	0.19
**FLT3**	FLT3 neg	24.4%[19.8-29.4]	14.4%[10.9-18.4]	61.2%[55.4-66.4]	65.8%[60-70.9]	46.6%[41.1-52]
	FLT3 pos	27%[19.6-34.9]	11.7%[6.8-18.2]	61.3%[52.1-69.2]	65.9%[56.4-73.8]	38.8%[30.3-47.3]
	P value	0.08	0.32	0.54	0.85	**0.026**
**NPM1**	NPM1 neg	24.7%[20.1-29.5]	13.8%[10.4-17.6]	61.5%[56-66.6]	67%[61.5-72]	45.4%[40.1-50.7]
	NPM1 pos	20.8%[13.6-29.2]	17.7%[10.9-25.9]	61.5%[51-70.3]	64.2%[53.2-73.3]	43.2%[33.3-52.8]
	P value	0.61	0.39	0.79	0.65	0.75

Supplementary table 2: Outcome among patients with de novo AML (n=584) and patients with intermediate-risk cytogenetics (n= 510) as of *RUNX1* mutational status

		2 years				
		**RI**	**NRM**	**LFS**	**OS**	**GRFS**
**De novo AML**						
**Overall Outcome**		22.1% [95% CI: 18.4-26.2]	15.5% [95% CI: 12.4-18.9]	62.4% [95% CI:57.6-66.7]	67.4% [95% CI: 62.7-71.7]	45.9% [95% CI: 41.2-50.5]
**RUNX1**	Absent	23.4%[19-28.1]	15.6%[12.1-19.6]	61%[55.5-66]	66.1%[60.6-71]	45%[39.7-50.2]
	Present	18%[11.4-25.9]	15.5%[9.1-23.4]	66.5%[56.2-74.9]	71.2%[60.6-79.5]	48.6%[38.9-57.7]
	P value	0.22	0.84	0.24	0.25	0.44
**Intermediate-risk cytogenetics**						
**Overall Outcome**		21.4% [95% CI: 17.4-25.7]	14.1% [95% CI: 10.9-17.6]	64.6% [95% CI: 59.5-69.2]	70.3% [95% CI: 65.4-74.8]	48.9% [95% CI: 43.9-53.8]
**RUNX1**	Absent	21.5%[17-26.4]	14.3%[10.7-18.4]	64.2%[58.4-69.4]	69.1%[63.3-74.2]	49.4%[43.7-54.9]
	Present	21.5%[13.2-31.1]	13.6%[7.5-21.5]	64.9%[53.4-74.3]	73.9%[63.1-82]	46.6%[35.8-56.7]
	P value	0.51	0.55	0.3	0.3	0.72

Supplementary table 3: Exploratory analysis of further, genetically defined subgroups

		2 years				
		**RI**	**NRM**	**LFS**	**OS**	**GRFS**
**ASXL1**	Absent **n=191**	20.6% [14.4-27.5]	17.1% [11.7-23.4]	62.3% [53.9-69.6]	63.6% [54.7-71.2]	45.6% [37.5-53.3]
	Present **n=67**	29.2% [17.1-42.4]	11.8% [5.1-21.6]	59% [44.1-71.2]	64.4% [49.4-76.1]	37.2% [23.5-50.9]
	P value	0.17	0.36	0.6	0.94	0.44
**RUNX1 / ASXL1**	RUNX1- ASLX1- **n=161**	21.5% [14.6-29.2]	18.2% [12.1-25.3]	60.4% [51-68.5]	61.8% [52-70.2]	44.9% [36.1-53.3]
	RUNX1- ASLX1+ **n=30**	21.5% [8.4-38.4]	21.8% [8.6-38.9]	56.7% [36.4-72.8]	58% [36.2-74.6]	30.9% [14.9-48.5]
	RUNX1+ ASLX1- **n=30**	16.1% [4.8-33.2]	11.7% [2.8-27.5]	72.2% [50.2-85.7]	73.5% [49.5-87.4]	49.4% [28.8-67.1]
	RUNX1+ ASLX1+ **n=37**	37.4% [17.1-57.8]	2.7% [0.2-12.3]	59.9% [36.5-77.1]	70.4% [48.3-84.4]	43.2% [22.3-62.5]
	P value (global)	0.35	0.15	0.5	0.6	0.36

Supplementary table 4: Multivariate analysis among patients with de novo AML (n=585) and patients with intermediate cytogenetics (n=510)

**De novo AML (n=584)**										
	**RELAPSE**		**NRM**		**LFS**		**OS**		**GRFS**	
**Multivariate analysis**	**HR (95% CI)**	**p value**	**HR (95% CI)**	**p value**	**HR (95% CI)**	**p value**	**HR (95% CI)**	**p value**	**HR (95% CI)**	**p value**
RUNX1 vs no	0.8 (0.44-1.44)	0.45	0.75 (0.37-1.53)	0.44	0.79 (0.5-1.25)	0.32	0.84 (0.52-1.35)	0.47	1.1 (0.78-1.56)	0.57
UD/Haplo vs MSD	1.39 (0.85-2.27)	0.19	2.62 (1.2-5.69)	**0.015**	1.75 (1.16-2.64)	**0.008**	2 (1.26-3.18)	**0.003**	1.33 (0.97-1.84)	0.078
Age (per 10y)	0.85 (0.72-1)	**0.049**	2.14 (1.57-2.92)	**< 0.0001**	1.11 (0.97-1.28)	0.14	1.27 (1.08-1.49)	**0.004**	1.02 (0.91-1.14)	0.71
Poor cyto vs interm.	1.84 (1.18-2.89)	**0.008**	1.38 (0.77-2.47)	0.28	1.68 (1.18-2.39)	**0.004**	1.57 (1.07-2.3)	**0.021**	1.58 (1.18-2.13)	**0.002**
FLT3 ITD	1.72 (1.11-2.65)	**0.015**	0.99 (0.55-1.81)	0.98	1.34 (0.95-1.89)	0.1	1.19 (0.82-1.75)	0.36	1.38 (1.04-1.84)	**0.025**
Female to male	0.9 (0.46-1.76)	0.76	2.11 (1.16-3.85)	**0.015**	1.34 (0.86-2.08)	0.19	1.44 (0.91-2.28)	0.12	1.07 (0.73-1.57)	0.73
RIC vs MAC	1.51 (0.96-2.39)	0.075	0.89 (0.5-1.59)	0.7	1.27 (0.89-1.82)	0.18	1.3 (0.88-1.93)	0.19	1.13 (0.83-1.54)	0.45
in vivo TCD	1.12 (0.74-1.69)	0.6	0.68 (0.4-1.14)	0.14	0.91 (0.66-1.25)	0.55	0.81 (0.57-1.14)	0.23	0.74 (0.55-0.99)	**0.042**
**Intermediate-risk cytogenetics (n=510)**										
	**RELAPSE**		**NRM**		**LFS**		**OS**		**GRFS**	
**Multivariate analysis**	**HR (95% CI)**	**p value**	**HR (95% CI)**	**p value**	**HR (95% CI)**	**p value**	**HR (95% CI)**	**p value**	**HR (95% CI)**	**p value**
RUNX1 vs no	1.11 (0.64-1.94)	0.71	0.56 (0.24-1.33)	0.19	0.88 (0.56-1.39)	0.59	0.84 (0.5-1.39)	0.49	1.19 (0.82-1.72)	0.35
UD/Haplo vs MSD	0.96 (0.56-1.63)	0.87	3.07 (1.18-8.02)	**0.022**	1.42 (0.9-2.23)	0.13	1.59 (0.96-2.63)	0.074	1.16 (0.81-1.66)	0.43
Age (per 10y)	0.86 (0.71-1.03)	0.11	2.24 (1.56-3.2)	**< 0.0001**	1.13 (0.96-1.33)	0.13	1.29 (1.08-1.55)	**0.006**	1.03 (0.91-1.17)	0.62
Secondary vs de novo AML	1.73 (0.98-3.05)	0.058	0.37 (0.13-1.06)	0.065	1.11 (0.68-1.8)	0.68	0.91 (0.52-1.59)	0.74	1 (0.65-1.52)	0.99
FLT3 ITD	1.63 (1.03-2.59)	**0.038**	1.18 (0.63-2.19)	0.61	1.42 (0.98-2.05)	0.061	1.35 (0.91-2.02)	0.14	1.57 (1.16-2.12)	**0.003**
Female to male	0.73 (0.36-1.5)	0.4	2.25 (1.17-4.34)	**0.015**	1.17 (0.73-1.88)	0.5	1.33 (0.81-2.18)	0.27	1.06 (0.71-1.59)	0.76
RIC	1.73 (1.02-2.94)	**0.041**	0.87 (0.46-1.66)	0.68	1.33 (0.89-1.97)	0.16	1.22 (0.8-1.88)	0.36	1.16 (0.82-1.64)	0.41
in vivo TCD	1.07 (0.65-1.76)	0.8	0.78 (0.44-1.38)	0.39	0.95 (0.66-1.37)	0.79	0.87 (0.59-1.29)	0.5	0.83 (0.58-1.18)	0.3
